# Perspectives and Values of Dental Medicine Students Regarding Domestic Violence

**DOI:** 10.3390/medicina57080780

**Published:** 2021-07-30

**Authors:** Oana-Maria Isailă, Sorin Hostiuc, George-Cristian Curcă

**Affiliations:** 1 Department of Legal Medicine and Bioethics, Faculty of Dental Medicine, “Carol Davila” University of Medicine and Pharmacy, RO-020021 Bucharest, Romania; oana_maria.isaila@yahoo.com; 2 “Mina Minovici” National Institute of Legal Medicine, RO-042122 Bucharest, Romania; cgcurca@yahoo.com; 3 Department of Legal Medicine and Bioethics, Faculty of Medicine, “Carol Davila” University of Medicine and Pharmacy, RO-020021 Bucharest, Romania

**Keywords:** domestic violence, physician–patient relationship, dental medical students’ opinion

## Abstract

*Background and Objectives*: The purpose of this study is to evaluate dental medical students’ opinions concerning domestic violence from a social and medical standpoint and from the perspective of the moral values of the physician–patient relationship. *Materials and Methods*: We performed an observational study with 4- and 5-year dental medical students at the UMF “Carol Davila” in Bucharest from October 2020–May 2021, using a questionnaire containing 20 items on domestic violence (DV). The questionnaire was uploaded online on the e-learning platform where the students have access. To collect the data, we used Microsoft Excel 365, and the statistical analysis was performed using Jamovi. *Results*: Of the 600 students enrolled, 415 answered the questionnaire, the answering rate being 69.16%. A total of 215 (53.1%) personally knew victims of DV, 4 (1.0%) considered that violence within a couple is necessary for certain situations, 401 (99.0%) considered that domestic violence is a fundamental problem in today’s society, and 170 (41.5%) felt that in domestic violence situations, the blame lies solely with the partner who resorts to physical violence. Regarding the role of the physician, 220 (56%) considered that the physician should breach confidentiality and report cases when patients state they are a victim of DV, 337 (88.2%) thought that free medical treatment should be provided for DV victims who have a dire financial situation, and 212 (56.7%) considered that victims of DV are non-compliant patients. *Conclusions*: Domestic violence is a phenomenon well-known to stomatology students, which creates the premise of an excellent physician–patient relationship with them, aiding in proper management of ethical issues such as a potential need to breach confidentiality or evaluate the potential conflicts between autonomy and beneficence.

## 1. Introduction

The United Nations defines domestic violence as “a pattern of behavior in any relationship that is used to gain or maintain power and control over an intimate partner. Abuse is physical, sexual, emotional, economical or psychological actions or threats of actions that influence another person. This includes any behaviors that frighten, intimidate, terrorize, manipulate, hurt, humiliate, blame, injure, or wound someone” [1]. According to the WHO, domestic violence represents “any behavior within an intimate relationship that causes physical, psychological or sexual harm to those in the relationship, including acts of physical aggression, sexual coercion, psychological abuse and controlling behaviors [2].”

Domestic violence can be unidirectional when only one partner is the aggressor, or bidirectional—mutual violence within a couple, a situation in which the victim is also an aggressor [3,4,5]. According to the study conducted by Melander et al., depressive persons and persons who consume alcohol are more likely to be both a victim and an aggressor within the couple [5]. Previous studies have shown that when the woman is the aggressor, she may act aggressively toward the violent partner to defend herself, thus mimicking bidirectional violence [6,7,8].

The consequences of domestic violence can be physical, such as traumatic injuries, chronic pain, chronic diseases, sexually transmitted diseases, pregnancy, miscarriage/abortion, sexual dysfunction [9]; psychological, such as depression, suicidal ideation, the decrease in the level of satisfaction with life [10], anxiety, sleep disorders, post-traumatic stress disorder, low self-esteem, somatization [9]; or behavioral [9]. Studies performed on traumatic injuries of dental interest resulting from domestic violence showed fractures of the incisors to have the highest prevalence [11,12]. Additionally, DV victims have poor oral health due to improper hygiene, altered periodontal status, dental loss, and dental fractures [13]. Other lesions recognizable by the dental practitioner potentially suggest domestic violence are face bruises, cervical bruises, and abrasions due to manual/nose compression, bruising of the palatal arch, facial fractures, bitemark injuries, and tears lingual frenulum, or injuries on the upper limbs [14].

The most critical factors for domestic violence include a history of violence during childhood, alcohol use, a low social and professional status of the woman [15], drug abuse, a low education level of either the victim or the perpetrator, or a history of psychiatric disorders [16]. The leading causes of domestic violence, after Worden, can be based on individual factors (substance abuse, history of violence in the family, adultery/jealousy, psychiatric pathology, dominant personality), family factors (communication problems, relationship problems, stress, coming from an abusive family), macro-factors (low economic status, problems in the workplace or lack of employment) and other factors, such as lack of education, social isolation, not respecting the norms of society [17]. Protection factors are the relational and social ones, represented by restrictive legislation, social/community support, resources, and services offered by specialized structures [18] to provide victims with increased self-esteem, social integration, or optimal access to medical care [19].

As dental medicine students, who are future dental practitioners, will have patients who are victims of domestic violence, analyzing their perspectives and attitudes towards this phenomenon helps assure an adequate education and ensure an optimal response to domestic violence cases. The purpose of this study is to evaluate dental medical students’ opinions concerning domestic violence from a social and medical standpoint and the perspective of the moral values of the physician–patient relationship.

## 2. Materials and Methods

We performed an observational study with 4- and 5-year dental medical students at the UMF “Carol Davila” in Bucharest from October 2020–May 2021, using a questionnaire containing 20 items on domestic violence (DV). The questionnaire was uploaded online on the e-learning platform, where the students have access. To collect the data, we used Microsoft Excel 365, and the statistical analysis was performed using Jamovi.

The study IRB outcome code is 972/26.01.2021. The questionnaire was approved by the institutional ethics board, validated, and uploaded online on the e-learning platform, to which all dental students had access to online classes at the discipline. The questionnaire was optional and anonymous, this being the reason for which no demographic data was requested.

The first seven questions (1–7) targeted the quantification of the severity of this phenomenon using case vignettes in which answers were evaluated using a four-point scale, using the following potential answers: it is not a case of domestic violence; it is a case of slight domestic violence; it is a case of moderate domestic violence; it is a case of severe domestic violence. The following 10 questions (8–18) were dichotomous, with yes or no answers and targeted aspects concerning the importance of this phenomenon and the physician’s role in the relationship with the patient who is also a DV victim. Finally, the last two questions were multiple-choice and evaluated the media coverage and the potential causes of this phenomenon.

The data extracted from the questionnaire was collected using Excel 365, and the resulting database was processed with Jamovi v 1.2.27 (Topeka, KS, USA). We used descriptive statistics to evaluate the results obtained from the questionnaire.

## 3. Results

Of the 600 students, 415 answered the questionnaire; thus, the response rate was 69.16%. As there was no mandatory answer, some students only responded partially to the questionnaire. The responses to the case vignettes are summarized in Table 1. Briefly, most respondents correctly identified gross DV cases, even if they were not physical, directed toward the spouse. Bidirectional DV was harder to identify (answer to Q1), as was economic (Q3) or social (Q5).

More than half of the respondents personally knew victims of domestic violence (215, 53.1%). Only four respondents (1.0%) considered domestic violence necessary in some instances. Most considered domestic violence a critical problem in today’s society (401 respondents, 99.0%).

Almost half of the respondents (170, 41.5%) considered that, in domestic violence, the partner who resorts to physical violence is the only one to blame (see Table 2).

Regarding the physician’s role in recognizing this phenomenon, 257 (65.6%) considered that the physician must ask any patient about the existence of eventual conflicts in the family environment when there are physical signs potentially suggesting DV. Most respondents (323, 81.4%) did not consider that the physician should report the case to state authorities without discussing it with the patient first. More than half (212, 56.7%) believed that victims of DV are non-compliant patients, 220 (56%) thought that the physician should breach confidentiality and report the case when the patient affirms they are a victim of DV, 337 (88.2%) thought that free medical treatment should be offered to victims of DV who have low financial status, 182 (49.1%) considered that women who are victims of DV are discriminated on the assumption that their symptoms are imaginary, and 178 (48.1%) feel that men who are victims of DV are believed to exaggerate their symptoms. (see Table 3).

Regarding the attitude of mass media’s towards domestic violence, 241 (58.4%) respondents considered that cases of domestic violence are shown with the primary purpose to increase ratings, 104 (25.2%) believed these cases are presented to help raise awareness and to stop the phenomenon, and 68 (16.5%) considered that cases of domestic violence are not frequently showcased.

**Figure 1:**
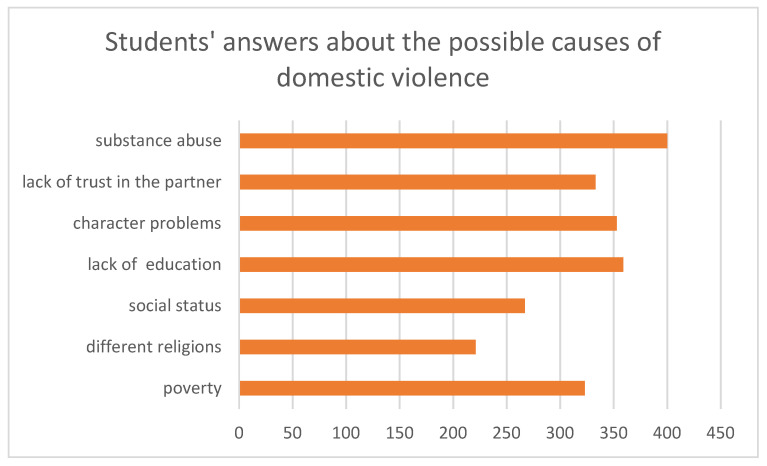
Students’ answers about the possible causes of domestic violence.

Regarding the students’ answers about the possible causes of domestic violence, they considered them being as follows: poverty, 323 (77.8%); different religions, 221 (53.3%); social status, 267 (64.3%); lack of education, 359 (86.5%); character problems, 353 (85.1%); lack of trust in the partner, 333 (80.2%); substance abuse, 400 (96.4%). See Figure 1 for details.

## 4. Discussion

Our study evaluated future dental practitioners’ perception of domestic violence, emphasizing the severity of its types and the physician’s role. 

The first seven questions evaluated the recognition and evaluation of the degrees of DV. From the received answers, students understood the clear-cut forms of domestic violence (verbal and physical). Still, they were less aware of the types that can be ambivalent and contextually arguable (social and economical).

The next items from the questionnaire have shown that future physicians know the significance and consequences of this phenomenon at both a micro level (families) and macro-level (societies). Regarding the physician’s role in recognizing and managing DV, most respondents considered DV victims non-compliant patients, which emphasizes the need for a personalized approach of the victim within the physician–patient relationship. McCloskey et al. have shown that for women who are victims of domestic violence, especially if they have a lower financial status and a low education level, the abusive partner stopped them from seeking medical assistance and, as a result, it generated deficits regarding proper access to medical care [20]. This can be seen as a significant control, affecting the autonomy of the victim. Seeking medical assistance by DV victims is often completed in secrecy (when the abuser is not home or sleeping). This emphasizes three main aspects that must be taken into account. First, emergency medical and social care for these patients should be given whenever possible, even outside regular hours. This approach is based on the virtues of loyalty—the physician should prioritize their patients, especially those that are particularly vulnerable, whenever the need arises and benevolence—assisting a person in need more than the physician is obligated is within their professional duties. The second aspect is the need for a more personalized approach to confidentiality that must be vigorously enforced with the family and friends of the patient but also breached toward relevant state agents (such as social workers or even the criminal system) whenever the physician believes there is an inherent risk for the patient [21]. The more robust type of confidentiality toward the family and friends is generated by potential (even if unwilling) disclosures about the visit to the medical office directed toward the perpetrator or somebody close to him. Therefore, physicians should not allow the presence of a third party when issues regarding DV are discussed with the patient, nor should they disclose such information to third parties, even if the patient accepts, unless they consider the involvement of these third parties indispensable for the management of the patient. Additionally, physicians should always inform the patient about the need to keep secrecy about the visit to the perpetrator (including issues such as hiding the receipt for the consultation or the medical letter containing details about the medical visit), as they can generate a new aggressive event. If, toward relatives and friends of the family, the disclosure should be minimized (even with the patient’s agreement), concerning social workers and other persons potentially helpful in managing acts of domestic violence, disclosure should be allowed, based on the duty to warn. For example, in Romania, a specific law provides anybody who suspects an act of domestic violence (medical workers included) to inform relevant state authorities [22]. The mandatory reporting of DV cases has been a highly controversial topic in the scientific literature throughout the years. For example, Antle et al. found DV victims to prefer disclosure for them and other patients in similar situations [23]. On the other side, Colter and Chez found that DV victims considered mandatory reporting useful only for other DV victims, but not if they were the subject [24]. In our study, most respondents (56.0%) agreed that when the physician is confronted with a DV victim, they should breach confidentiality and report the case. The third issue that has to be considered is the need for a particular type of physician–patient relationship. As the patients are often in shock or show increased anxiety levels, a purely informative model of the physician–patient relationship is not advisable, as the decisional capacity is often temporarily decreased. However, a paternalist model should never be applied in these cases, as it might reinforce some feelings generated by the aggressor (who is often dominant in relation with the victim). From our clinical experience, we recommend an interpretative approach to the professional relationship [25], which allows a translation of the actual wants and needs of the patient while maximizing its autonomy.

Most responders (88.2%) considered that free medical treatment would be helpful for victims of domestic violence who are in a dire financial situation. A lack of financial resources is known to be positively linked with increased DV. Therefore, from a Rawlsian perspective [26], providing free medical care to DV victims is the right act, this measure being able to increase their addressability to medical care but also (indirectly) to social, psychological, and judicial systems that could aid in limiting aggressive behaviors [27].

Regarding the role of mass media, most respondents (58.4%) considered that exposing domestic violence cases has the purpose of increasing ratings. Only 25.2% believed that increased media exposure might raise awareness and prevent this phenomenon. This result shows that mass media does not seem to have a preventive approach to DV but rather uses it for financial purposes.

Similar results regarding the inefficiency of mass media in preventing DV have been shown by Maquibar et al. [28].

Most responders (96.4%) considered substance abuse to be a main factor in generating DV. Alcohol and drug abuse are known to increase aggressive behavior in general [29] and in a domestic context in particular [30].

Dentists are less aware of DV, as shown by other published studies. For example, Love et al. found that most dentists never checked if their patients were victims of domestic violence and, in general, have intervened to a very little extent to help in cases of domestic violence. Among the obstacles they have encountered were the presence of the spouse or the child of the victim during the consultation, the embarrassment to ask, and a lack of instruction in this sense. It has been found that respondents who benefitted from adequate training were more determined to verify this phenomenon and intervene [31], this being one of the main reasons for which we have developed this questionnaire and brought it to the attention of medical dental students (together with a special course regarding the particularities of DV in dentistry, which is taught to 4-year medical dental students, in the legal medicine curriculum). Another study by Skelton et al. on the same subject indicated the need for dental practitioners to have supplemental instruction on the theme of domestic violence [32]. Drigeard et al. showed that most dental practitioners do not look for signs of potential domestic abuse in patients, only 36% declaring they have encountered DV, and 75.9% felt the need for additional training on the subject of domestic violence [33]. AlAlyani and Alshouibi concluded, in a similar study, that for dental practitioners, the following barriers exist in identifying patients as victims of domestic violence: a lack of instruction on this subject and an implicit hesitation of asking questions on this topic [34]. Mythri H. et al.’s study has found a low level of knowledge in dentists about DV and pointed out the need for additional education on this theme [35]. 

Studies performed on medical workers of other specialties have also shown significant barriers regarding the screening for domestic violence due to a lack of availability or time of the medical staff [36,37] or a lack of training in this respect [38,39].

The limitations of this study were the relatively small number of participants and the lack of their stratification according to demographic variables.

## 5. Conclusions

Domestic violence is a phenomenon well known to stomatology students, which creates the premise of an excellent physician–patient relationship with them, aiding in proper management of ethical issues such as a potential need to breach confidentiality or evaluate the potential conflicts between autonomy and beneficence.

## Figures and Tables

**Table 1:** Answers to the first seven questions.

Question (Number & Statement)	It’s Not DV	It’s a Slight DV	It’s a Moderate DV	It’s a Severe DV
Q1. It is a case of DV when the partners shout at each other?	133 (32.1%)	183 (44.2%)	86 (20.8%)	12 (2.9%)
Q2 Is cheating a form of DV?	275 (66.4%)	43 (10.4%)	52 (12.6%)	44 (10.6%)
Q3. A husband refusing to give money to his wife to pay the household utilities a type of DV?	189 (45.9%)	107 (26.0%)	86 (20.9%)	30 (7.3%)
Q4. Forgetting the spouse’s birthday is a form of DV?	383 (92.3%)	21 (5.1%)	9 (2.2%)	2 (0.5%)
Q5. A woman forbidding her spouse to go to a football match is a form of DV?	183 (44.2%)	166 (40.1%)	55 (13.3%)	10 (2.4%)
Q6. A man being aggressive with pets in the presence of his partner is a form of DV?	111 (26.7%)	70 (16.9%)	127 (30.6%)	107 (25.8%)
Q7. If the man does not pay the bill at a restaurant, is it a form of DV?	384 (92.5%)	20 (4.8%)	7 (1.7%)	4 (1.0%)

DV: domestic violence.

**Table 2:** The evaluation of the perception of the phenomenon.

Question (Number & Statement)	No	Yes
Q8. Do you personally know victims of domestic violence?	190 (46.9%)	215 (53.1%)
Q9. Do you consider violence within a couple necessary in certain situations?	402 (99.0%)	4 (1.0%)
Q10. Do you consider domestic violence a critical problem in today’s society	5 (1.2%)	401 (98.8%)
Q11. Do you consider that in domestic violence, the only responsible person is the one who resorts to physical violence?	222 (56.61%)	170 (43.4%)

**Table 3:** Perception of the physician’s role in the context of the domestic violence phenomenon.

Question (Number & Statement)	No	Yes
Q12. Do you consider that the physician should ask any patient about eventual conflicts within the family?	135 (34.4%)	257 (65.6%)
Q13. Suppose a person with traumatic lesions to the face and upper limbs visits the physician. Should the physician assume that the patient is a victim of domestic violence and breach confidentiality by notifying the competent authorities without discussing beforehand with the patient?	323 (81.4%)	74 (18.6%)
Q14. Do you consider victims of domestic violence non-compliant?	162 (43.3%)	212 (56.7%)
Q15. If a person claiming they were assaulted by their spouse visits the physician, should the physician breach confidentiality and report the case?	173 (44.0%)	220 (56.0%)
Q16. Do you consider that low-income patients who are victims of domestic violence should benefit from free medical treatment?	45 (11.8%)	337 (88.2%)
Q17. Do you consider that women who are victims of domestic violence are discriminated against when they go to the physician because they are simulating their symptoms?	189 (50.9%)	182 (49.1%)
Q18. Do you consider that men who are victims of domestic violence are discriminated against when they go to the physician because they are highly exaggerating their symptoms?	192 (51.9%)	178 (48.1%)

## Data Availability

Data supporting this results can be available online on elearnmed.ro only following an explicit request in this regard.
